# Express Method for Isolation of Ready-to-Use 3D Chitin Scaffolds from *Aplysina archeri* (Aplysineidae: Verongiida) Demosponge

**DOI:** 10.3390/md17020131

**Published:** 2019-02-22

**Authors:** Christine Klinger, Sonia Żółtowska-Aksamitowska, Marcin Wysokowski, Mikhail V. Tsurkan, Roberta Galli, Iaroslav Petrenko, Tomasz Machałowski, Alexander Ereskovsky, Rajko Martinović, Lyubov Muzychka, Oleg B. Smolii, Nicole Bechmann, Viatcheslav Ivanenko, Peter J. Schupp, Teofil Jesionowski, Marco Giovine, Yvonne Joseph, Stefan R. Bornstein, Alona Voronkina, Hermann Ehrlich

**Affiliations:** 1 Institute of Physical Chemistry, TU Bergakademie-Freiberg, Leipziger str. 29, 09559 Freiberg, Germany; cjtk1991@web.de; 2 Institute of Chemical Technology and Engineering, Faculty of Chemical Technology, Poznan University of Technology, Berdychowo 4, 61131 Poznan, Poland; soniazolaks@gmail.com (S.Ż.-A.); tomasz.g.machalowski@doctorate.put.poznan.pl (T.M.); teofil.jesionowski@put.poznan.pl (T.J.); 3 Institute of Electronics and Sensor Materials, TU Bergakademie Freiberg, Gustav Zeuner Str. 3, 09599 Freiberg, Germany; iaroslavpetrenko@gmail.com (I.P.); yvonne.joseph@esm.tu-freiberg.de (Y.J.); 4 Leibnitz Institute of Polymer Research Dresden, 01069 Dresden, Germany; tsurkan@ipfdd.de; 5 Clinical Sensoring and Monitoring, Department of Anesthesiology and Intensive Care Medicine, Faculty of Medicine, Technische Universität Dresden, 01307 Dresden, Germany; roberta.galli@tu-dresden.de; 6 Institut Méditerranéen de Biodiversité et d’Ecologie (IMBE), CNRS, IRD, Aix Marseille Université, Avignon Université, Station Marine d’Endoume, 13003 Marseille, France; alexander.ereskovsky@imbe.fr; 7 Department of Embryology, Faculty of Biology, Saint-Petersburg State University, 19992 Saint-Petersburg, Russia; 8 Institute of Marine Biology, University of Montenegro, 85330 Kotor, Montenegro; rajko.mar@ucg.ac.me; 9 Institute of Bioorganic Chemistry and Petrochemistry, National Academy of Science of Ukraine, Murmanska Str., 1, 02094 Kyiv, Ukraine; smolii@bpci.kiev.ua (O.B.S.); lmuzychka@rambler.ru (L.M.); 10 Institute of Clinical Chemistry and Laboratory Medicine, University Hospital Carl Gustav Carus, Faculty of Medicine Carl Gustav Carus, Technische Universität Dresden, 01307 Dresden, Germany; Nicole.bechmann@uniklinikum-dresden.de; 11 Department of Invertebrate Zoology, Biological Faculty, Lomonosov Moscow State University, 119992 Moscow, Russia; ivanenko.slava@gmail.com; 12 Naturalis Biodiversity Center, 2332 Leiden, The Netherlands; 13 Institute for Chemistry and Biology of the Marine Environment, University of Oldenburg, Carl-von-Ossietzky-Straße 9-11, 26111 Oldenburg, Germany; peter.schupp@uni-oldenburg.de; 14 Department of Sciences of Earth, Environment and Life, University of Genoa, Corso Europa 26, 16132 Genova, Italy; mgiovine@unige.it; 15 Department of Internal Medicine III, University Hospital Carl Gustav Carus, Technische Universität Dresden, 01307 Dresden, Germany; Stefan.bornstein@uniklinikum-dresden.de; 16 Diabetes and Nutritional Sciences Division, King’s College London, London WC2R 2LS, UK; 17 National Pirogov Memorial Medical University, Vinnytsya, Department of Pharmacy, Pirogov str. 56, 21018, Vinnytsia, Ukraine; algol2808@gmail.com

**Keywords:** chitin, marine sponges, scaffolds, *Aplysina archeri*, express method, bromotyrosines, crude oil, blood, methylene blue

## Abstract

Sponges are a valuable source of natural compounds and biomaterials for many biotechnological applications. Marine sponges belonging to the order Verongiida are known to contain both chitin and biologically active bromotyrosines. *Aplysina archeri* (Aplysineidae: Verongiida) is well known to contain bromotyrosines with relevant bioactivity against human and animal diseases. The aim of this study was to develop an express method for the production of naturally prefabricated 3D chitin and bromotyrosine-containing extracts simultaneously. This new method is based on microwave irradiation (MWI) together with stepwise treatment using 1% sodium hydroxide, 20% acetic acid, and 30% hydrogen peroxide. This approach, which takes up to 1 h, made it possible to isolate chitin from the tube-like skeleton of *A. archeri* and to demonstrate the presence of this biopolymer in this sponge for the first time. Additionally, this procedure does not deacetylate chitin to chitosan and enables the recovery of ready-to-use 3D chitin scaffolds without destruction of the unique tube-like fibrous interconnected structure of the isolated biomaterial. Furthermore, these mechanically stressed fibers still have the capacity for saturation with water, methylene blue dye, crude oil, and blood, which is necessary for the application of such renewable 3D chitinous centimeter-sized scaffolds in diverse technological and biomedical fields.

## 1. Introduction

Chitin is composed of β(1,4)-linked N-acetyl-glucosamine units and represents the most abundant structural polysaccharide of invertebrates, including such marine phyla as sponges, corals, annelid worms, mollusks, and arthropods [1]. This biopolymer is mostly found in the skeletal structures of invertebrates, and plays a crucial role in their rigidity, stiffness, and other mechanical properties. Chitin is recognized as one of the universal templates in biomineralization, with respect to both biocalcification and biosilicification [2]. Chitin has been found in diverse organisms as layers—in mollusks’ shells [3], glass sponges’ skeletal frameworks [4], and spicules [5]—or as three-dimensional (3D) constructs: cuticles of crustaceans [6], skeletons of certain demosponges [7,8,9,10], and especially within mineralized tissues [11]. Very often, chitin is found in association with such organic compounds as proteins, lipids, pigments, and other polysaccharides [12]. Thus, the efficient extraction of pure chitin from the marine sources listed above is strongly dependent on methodological progress, which, however, is usually limited by the presence of corresponding mineral and foreign organic phases. Consequently, the industrial methods of chitin isolation most used in this case are based on chemical treatments that enable depigmentation [13], hydrolysis of proteins, and demineralization of inorganic matter [14,15,16,17,18,19].

Industrial deproteinization is carried out using a base solution of KOH or NaOH (for review, see [20,21]). The effectiveness of this treatment depends on the ratio of shells to solution, the temperature and duration of the process, as well as the concentration of the base. Industrial methods also include a depigmentation (decolorization) step, which improves the color of the chitin. For this purpose, hydrogen peroxide or sodium hypochlorite solutions are commonly used [22].

Demineralization can be achieved using chelators (ethylenediamine tetraacetic acid-EDTA [23]), or various acids, most commonly diluted hydrochloric acid, acetic acid, or sulfuric acid (for examples, see [22,24,25]). To achieve complete dissolution of all the inorganic salts, the acid intake should be greater than the stoichiometric quantity of the corresponding mineral phases [26,27]. Furthermore, the penetration of solvent into the chitin-based matrix is strongly dependent on the particle size [28]. In the case of crustacean shells, due to the heterogeneity of the solid matrix and difficulties in removing calcium debris, larger volumes, or more concentrated acid solutions, have traditionally been used [24,29]. Demineralization at higher temperatures has also been reported [30]. Methods based on long-term demineralization (even up to several days) may cause degradation of the biopolymer [31]. Alternatively, deproteinization and demineralization can also be carried out using microorganisms and corresponding enzymatic treatment. It has been reported [32] that biological treatment leads to better results than chemical methods, as it preserves the structure of chitin. New data on methods for chitin isolation, including applications of ionic liquids and eutectic solvents, have recently been published by Berton et al. [33], El Knidri et al. [34], Tokatlı & Demirdöven [35], Dun et al. [36], Saravana et al. [37], Castro et al. [38], Huang et al. [39], and Hong et al. [40].

**Figure 1:**
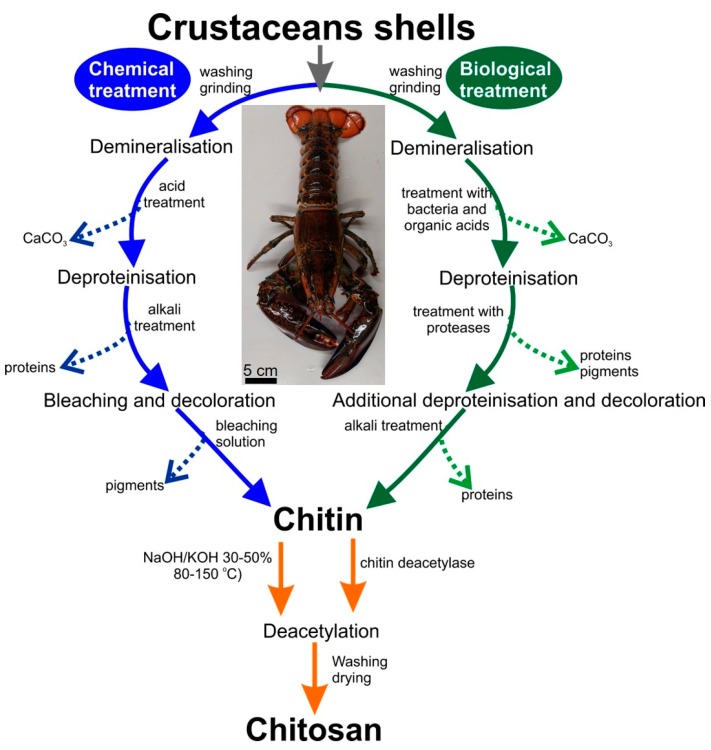
Schematic view of the standard biological and chemical approaches to the isolation of chitin-based biomaterials of crustacean origin.

A schematic overview of biological and chemical methods of chitin isolation for obtaining chitosan, following the example of crustacean sources based on the literature reports cited above, is presented in Figure 1.

Unlike chitin of crustacean origin, poriferan chitin has never been used as a source for chitosan. Besides spongin [41], chitin is one of the main structural biopolymers in demosponges with a 3D architecture, whose porous, fibrous network supports the sponges’ cells. Moreover, chitin is a key biological material for the stabilization of the sponges’ skeletons in marine habitats with strong ocean currents.

Since 2007, when chitin in the form of 3D scaffolds was isolated from selected demosponges for the first time [4], chitin has been verified in 17 marine species, mostly representing the order Verongiida [42,43].

**Figure 2:**
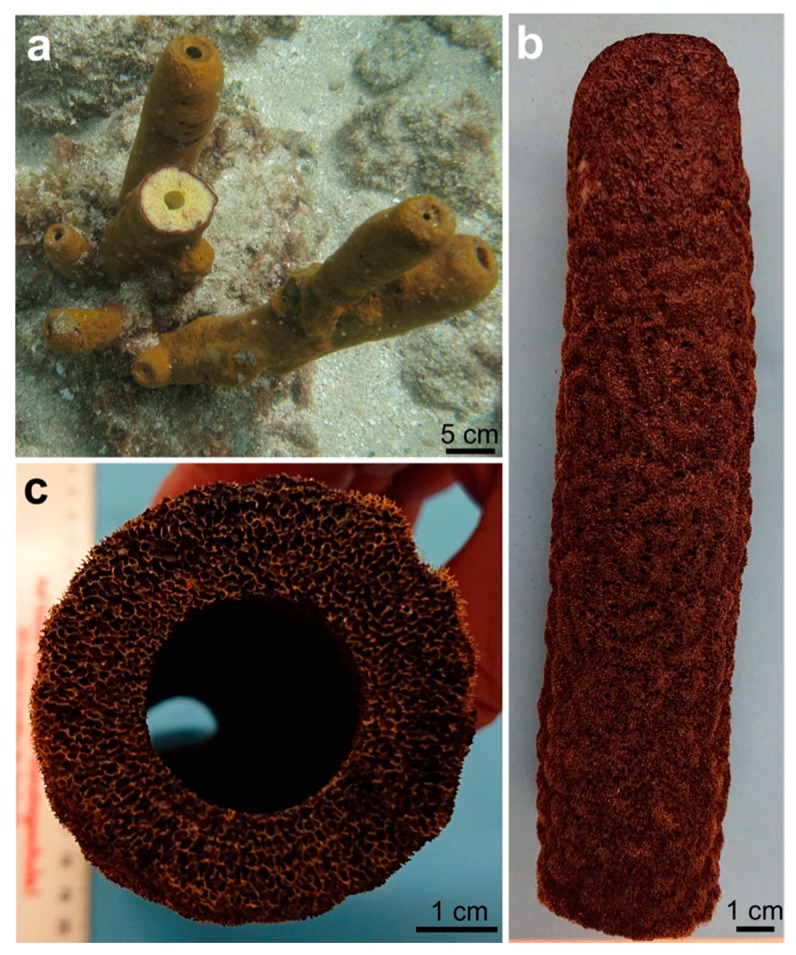
Underwater image (**a**) of the demosponge *A. archeri* shows the typical apical growth form of tube-like sponge bodies measuring up to 50 cm (or in some cases more than 2 m). A dried fragment of the sponge (**b**) remains pigmented and is hard enough to be cut using a metal saw (**c**). See also Figure 3a.

**Figure 3:**
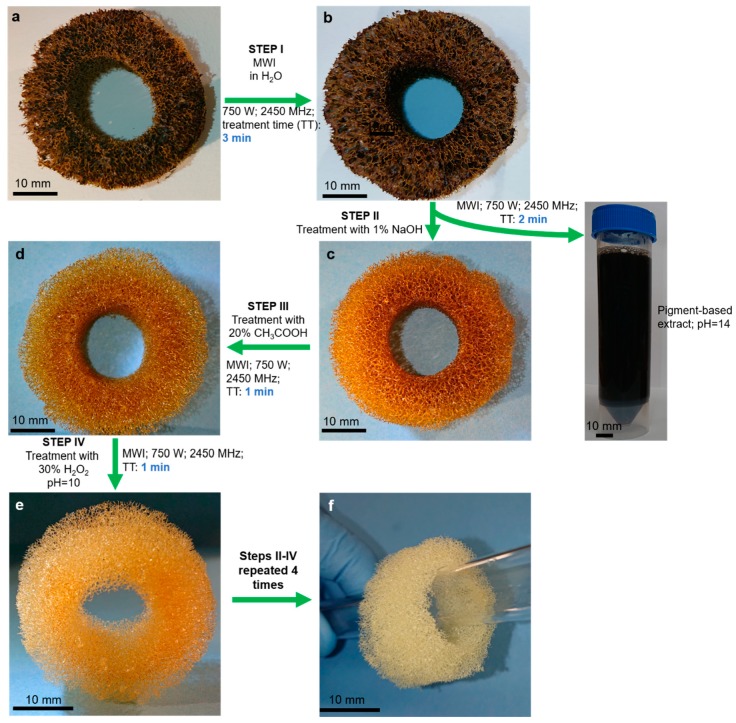
Schematic view of the microwave-assisted extraction of 3D chitin scaffold from a selected skeleton fragment (**a**) of the demosponge *A. archeri*. In step I, residual water soluble salts were removed by pretreatment of the sponge skeleton in distilled water under microwave irradiation (MWI) for 3 min. Afterwards, in step II (**b**), the sample was treated with 1% NaOH and irradiated with microwaves for 2 min. This procedure obtained both a 3D scaffold and a dark red-brownish pigment-containing extract (**c**). The 3D scaffold was carefully isolated and rinsed three times with distilled water to remove residual pigments. In step III, the 3D scaffolds were treated with 20% acetic acid under MWI for 1 min, and afterwards were washed in distilled water up to pH 6.8. In step IV (**d**,**e**), the sample was completely decolorized using 30% H_2_O_2_ at pH~10 under MWI for 1 min and washed with distilled water up to neutral pH. Steps II–IV were repeated four times to obtain a pure, colorless, ready-to-use 3D chitinous scaffold (**f**).

For the isolation of chitin from demosponges, a well-established method based on alternating alkaline (NaOH) and acidic (HF, HCl, CH_3_COOH) extraction steps, enabling the desilicification and decalcification of the mineralized sponge skeleton, has been used (for review, see [42,43]). Pigments, lipids and proteins are removed at the same time. In view of the large quantities of chemicals used and the time required by the individual extraction steps (up to 7 days), the method needs to be improved. In this study, we decided to use, for the first time, a complex approach that includes microwave irradiation (MWI) together with stepwise treatment using 1% sodium hydroxide, 20% acetic acid, and 30% hydrogen peroxide to isolate chitin from the tube-like skeleton of *Aplysina archeri* (Higgin, 1875) (phylum Porifera, class Demospongiae, Aplysineidae: order Verongiida, family Aplysinellidae) (Figure 2) [44]. An additional goal of the study was to develop an express isolation method (Figure 3), enabling the production of (i) crude extracts containing the pigments and (ii) ready-to-use 3D chitin scaffolds for practical applications in technology and biomedicine.

## 2. Results

### 2.1. Isolation and Identification of Chitin

The investigated sponge belongs to the family Aplysinellidae (order Verongiida), and hence is reported as a renewable source of chitin (for review, see [42,43]) as well as of the pharmaceutically relevant bromotyrosines [45]. The methodology is described in Figure 3. For comparative purposes, we carried out preliminary experiments on the isolation of chitin from the sponge fragments using the standard alkaline-based procedure, which takes up to 7 days (for details, see [9,10]). Using this approach, the naturally occurring mineralized skeletons of *A. archeri* became completely soft, colorless, and demineralized in a time of 114 h. However, the application of microwaves (750 W and 2450 MHz), as reported here, decreased the treatment time to only 55 min. The most time (33 min) was taken by the water rinsing procedures. The estimated chitin content in the skeletons of *A. archeri* is ~5% by weight.

**Figure 4:**
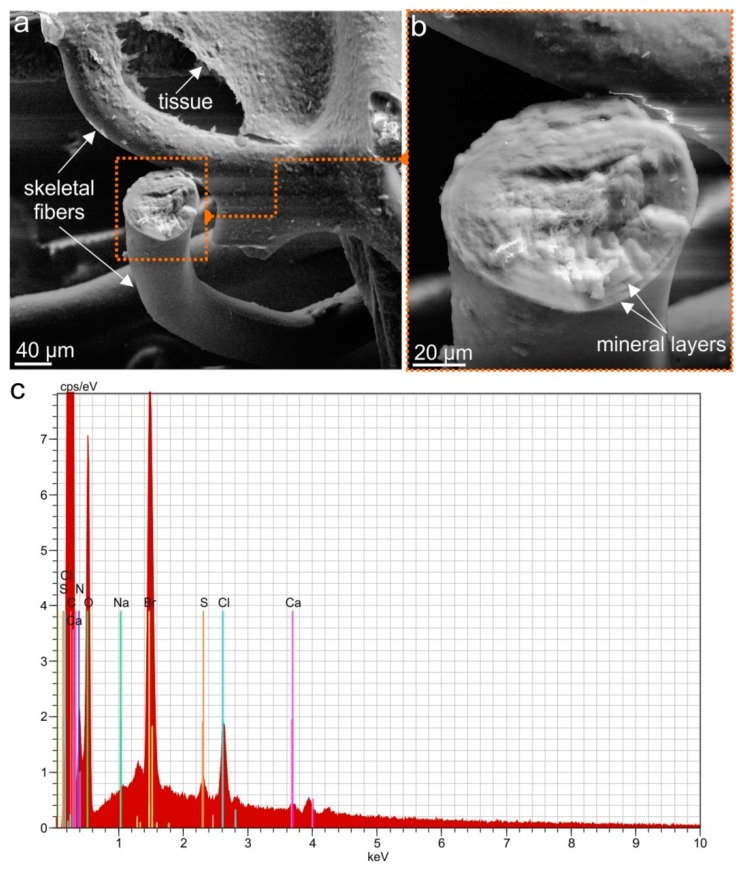
SEM microphotographs of the *A. archeri* skeletal fibers as collected (see Figure 3a,b) in different magnifications (**a**,**b**). EDX analysis of the fiber cross-section (**b**) reveals the presence of Ca, Cl, S and Br (**c**). This is similar to EDX data reported previously for naturally occurring skeletons of other verongiid demosponges (see [11]). The presence of bromine is likely determined by the localization of bromotyrosines within the chitinous layers of the skeletal fibers.

**Figure 5:**
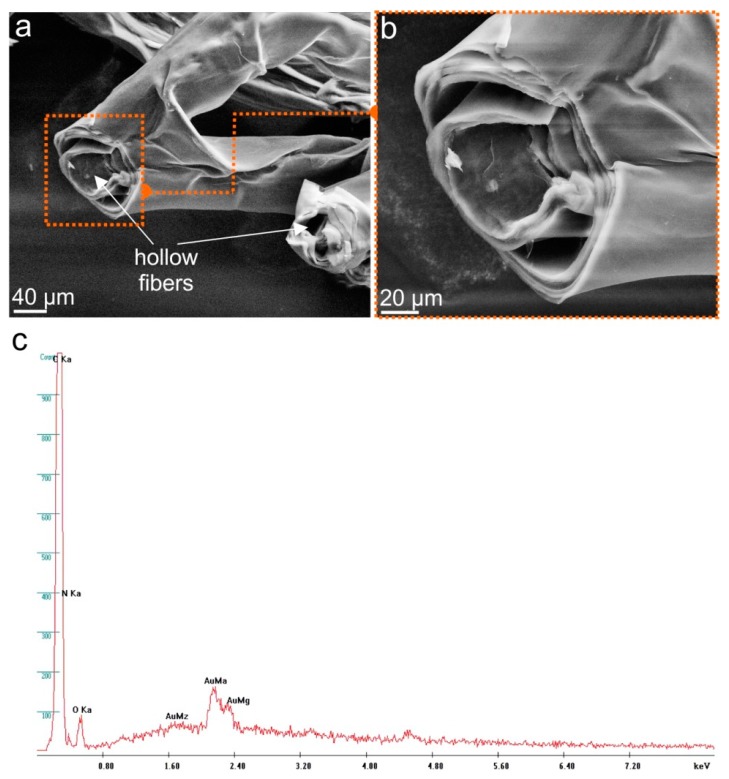
SEM imagery of the demineralized and depigmented tube-like skeletal fibers (see Figure 3f) obtained after microwave treatment (**a**,**b**). EDX analysis of these hollow structures (initially sputtered with Au) reveals the presence of C, N, and O only (**c**).

The microstructure of the demosponge skeletal fibers before (Figure 4a,b) and after isolation of the chitin scaffold (Figure 5a,b) was investigated using scanning electron microscopy (SEM). The recorded microphotographs display no visible changes in the microwave-stressed structure of the isolated material. Similar to the standard alkaline-based method for chitin isolation from demosponges using 5% NaOH at 37 °C [4,9,10], the new approaches lead to the preservation of the generic fibrous structure. The microphotographs of the fibers isolated from *A. archeri*, with the microwave approach also displaying the typical fibrous structure with concentric layers typical for microtubular chitin, previously reported in other representatives of the family Aplysinellidae [9,46]. The typical wrinkled structure (Figure 5a) due to the alkaline treatment occurs in chitinous fibers independent of microwave treatment, probably due to the very short time of microwave irradiation. EDX analysis of the naturally occurring skeletal structures of *A. archeri* (Figure 4c) and of the isolated chitin (Figure 5c) confirms the purity of the isolated biological material. The lack of characteristic elements in the isolated sample confirms that this proposed method is effective with respect to both demineralization and deproteinization.

After isolating completely colorless and mineral-free 3D scaffolds from selected fragments of the *A. archeri* skeleton (Figure 3f), we continued experiments in order to identify if either chitin or chitosan (due to the harsh treatment conditions with pH 14 and 95 °C) were present.

**Figure 6:**
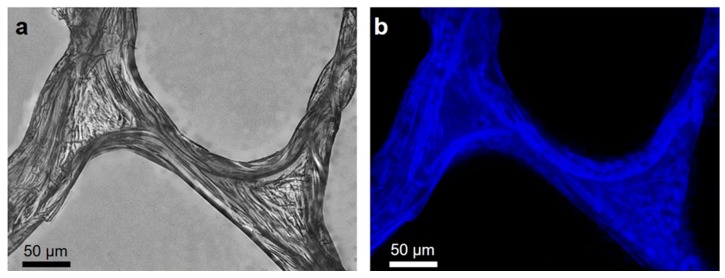
Light microscopy (**a**) and fluorescence microscopy (**b**) images of the selected fragment of a *A. archeri* scaffold after calcofluor white (CFW) staining. Intensive blue fluorescence remains measurable under a light exposure time of 1/3700 s.

The first step in identifying the isolated biological material as chitin or chitosan was calcofluor white (CFW) staining. On binding to polysaccharides, such as chitin, this fluorochrome emits a blue light. The microfibers of the scaffold isolated from *A. archeri* with the microwave-assisted method (Figure 3) show characteristic enhanced fluorescence after CFW staining (Figure 6). This result is similar to CFW-based poriferan chitin identification, as reported previously [4,8,9,10,42,43,46].

**Figure 7:**
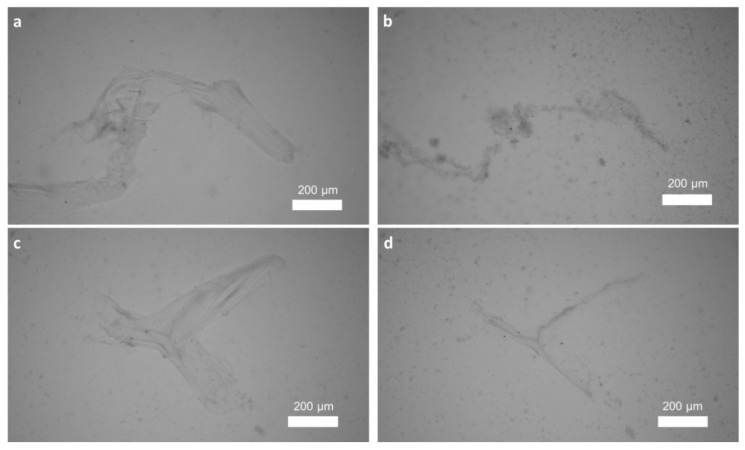
Chitinase test with chitin isolated from *A. archeri* using the standard method (see for details [9]) (**a**,**b**) and the microwave-based method (**c**,**d**). Images (**b**) and (**d**) were obtained after 4 h of incubation in chitinase solution.

Chitinase digestion is a well-recognized and highly specialized test to determine the chitinous nature of isolated scaffolds from diverse sponges. The enzyme catalyzes the endo-hydrolysis of *N*-acetyl-β-d-glucosamine-β-(1→4)-linkages. The activity of chitinase against microfibers isolated from the scaffolds being studied is shown in Figure 7.

**Figure 8:**
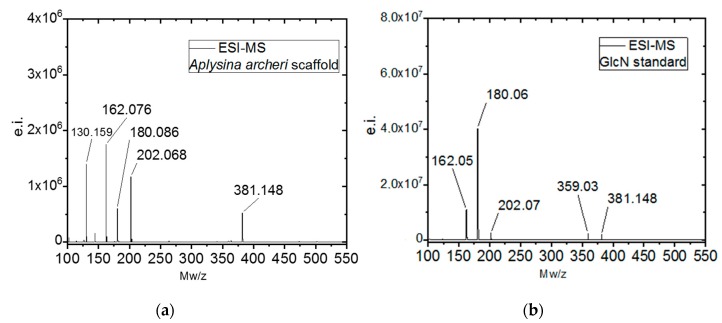
Electrospray-ionization mass spectroscopy (ESI-MS) spectra of (**a**) chitin from the scaffold of *A. archeri;* (**b**) a commercial (dGlcN) standard for comparison.

Additionally, we used electrospray ionisation mass spectrometry (ESI-MS) for identification of *N*-acetylglucosamine as a characteristic marker of the presence of chitin in unknown biological samples. Acetic hydrolysis of chitin by strong acids resulted in the formation of d-glucosamine (dGlcN), which can be easily visualized in ESI-MS spectroscopy. This method is a standard for chitin identification and has been used to identify chitin in various organisms [5,47] and fossil remains [48]. The ESI-MS spectrum of the *A. archeri* hydrolyzed scaffold, shown in Figure 8a, has five main ion peaks at *m*/*z* 130.16, 162.08, 180.09, 202.07 and 381.15. Four of the main peaks at *m*/*z* 162.08, 180.09, 202.07, and 381.15 are similar to those on the spectrum of the dGlcN standard (Figure 8b). These peaks correspond to dGlcN species of molecular ion (*m*/*z* 180.09), the specie with the loss of one H_2_O molecule [M − H_2_O + H]^+^ (*m*/*z* 162.08), the specie of sodium ion bound dGlcN monomer [M + Na]^+^ (*m*/*z* 202.07), and the noncovalent dimer [2M + Na]^+^ (*m*/*z* 381.15). A similar proton-bound GlcN covalent dimer with *m*/*z* = 359.17 was observed in the spectra. Together, these data indicate the high purity of the chitin in the *A. archeri* scaffold.

**Figure 9:**
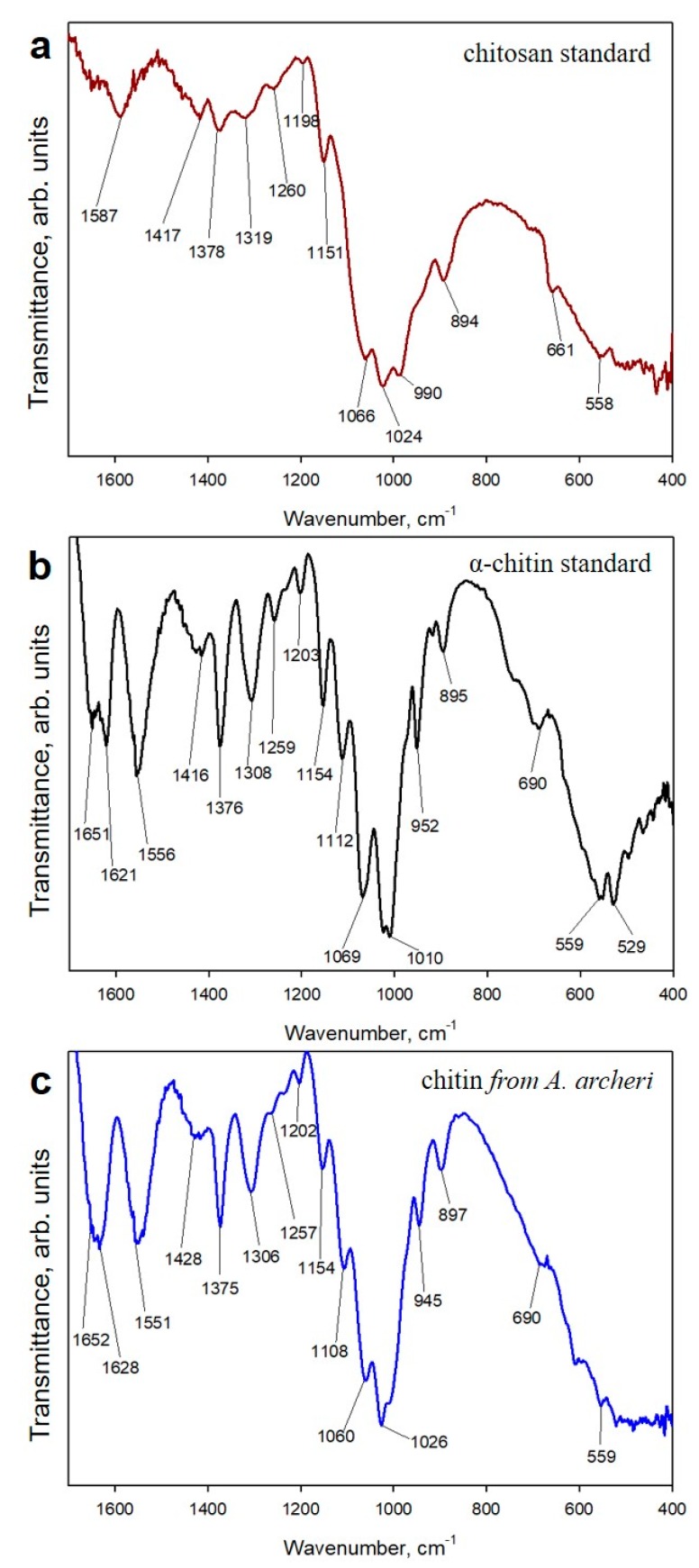
ATR-FTIR spectra of (**a**) chitosan, (**b**) α-chitin and (**c**) chitin isolated from *A. archeri* using the microwave-assisted approach (see Figure 3).

**Figure 10:**
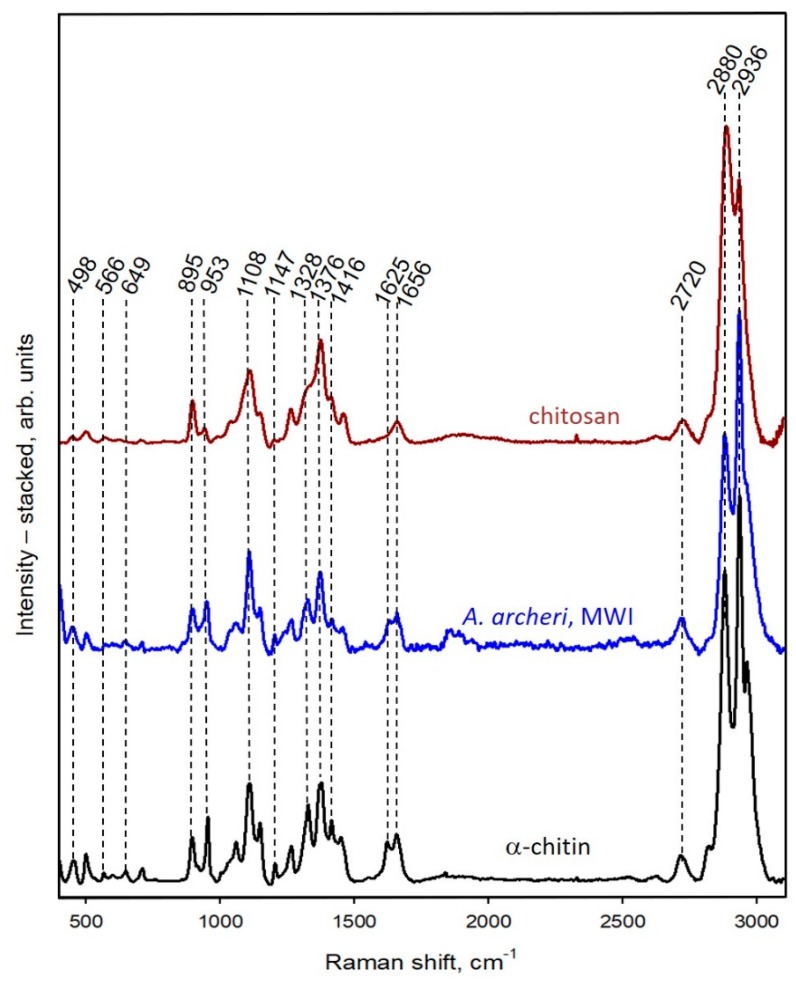
Raman spectra of chitosan standard (red line), α-chitin standard (black line), and chitin isolated from *A. archeri* demosponge using the microwave-assisted approach (blue line).

However, the analytical methods described above cannot provide an answer as to the possible transformation of chitin from *A. archeri* into chitosan under the conditions applied. Consequently, we decided to use both ATR-FTIR and Raman spectroscopy as highly sensitive methods to investigate this possibility. To exclude the possibility of deacetylation during the isolation of chitin with the microwave approach (see Figure 3), ATR-FTIR (Figure 9) and Raman spectra (Figure 10) of the scaffolds isolated from *A. archeri* are compared with the spectra of a chitosan standard and the α-chitin standard (Figure 9).

The ATR-FTIR spectrum, obtained for the scaffold isolated from *A. archeri*, shows a characteristically split amide I band at the positions 1651 and 1628 cm^−1^, associated with the stretching vibrations of the C=O bonds (Figure 9). This splitting of the amide I band is observed in the spectrum recorded for the α-chitin standard and is representative of this polymorph. It is observed due to the presence of stretching vibrations from intermolecular (C=O⋯HN) and intramolecular (C=O⋯HO(C6); C=O⋯HN) hydrogen bonds in the α-chitin chain [46,49,50]). These peaks are not visible in the spectrum of chitosan, because chitosan is a deacetylated derivative of chitin. Differences between the spectra recorded for the isolated scaffold, α-chitin standard and chitosan standard are also visible in the amide II band. For the isolated scaffold and α-chitin, the amide II band (N–H stretching vibrations) is recorded at the positions 1551 cm^−1^ and 1556 cm^−1^, whereas for chitosan it is shifted to the higher wavenumber, 1587 cm^−1^ (Figure 9). Another distinctive feature is the position of the amide III band (C–N stretching and N–H bending vibrations). In the spectra of *A. archeri* and α-chitin, this band is present at 1308 cm^−1^ and 1306 cm^−1^ respectively, while for chitosan the amide III band is shifted to a higher value (1318 cm^−1^), and its intensity is significantly lower. As an additional feature, the characteristic intense bands at 945 cm^−1^ and 950 cm^−1^, attributed to wagging vibrations of CH_x_, are observed only in the isolated scaffold and the α-chitin standard, respectively (Figure 9). Detailed analysis of the bands indicates that the spectra of the isolated scaffolds are very similar to those of the α-chitin standard. It can be concluded that the proposed microwave-assisted extraction, despite the very high temperature and alkaline pH, does not lead to the deacetylation of chitin, likely due to the very short time of microwave irradiation. Further studies should be carried out to optimize the isolation procedure based on microwave-assisted controlled surface deacetylation of tubular chitin scaffolds of poriferan origin.

The results of Raman analysis confirm those of ATR-FTIR. The Raman spectra of the scaffolds of *A. archeri* isolated using the microwave-assisted method exhibit good equivalence with the spectrum of α-chitin, and not with that of chitosan (Figure 10). The main differences are clearly visible in the amide I, amide II, and amide III regions.

### 2.2. Capillary Effect of 3D Chitin Scaffolds

We also investigated the properties of 3D microtubular sponge chitin, related to its function as a capillary system. Previously, we reported that due to their tubular structure, chitinous scaffolds from the demosponge *Aplysina aerophoba* swelled with zirconyl salt solution and could be used as liquid delivery constructs [51]. Here, we chose to use several different model liquids, such as crude oil, pig blood, and methylene blue dye, particularly due to the differences in their physicochemical properties.

**Figure 11:**
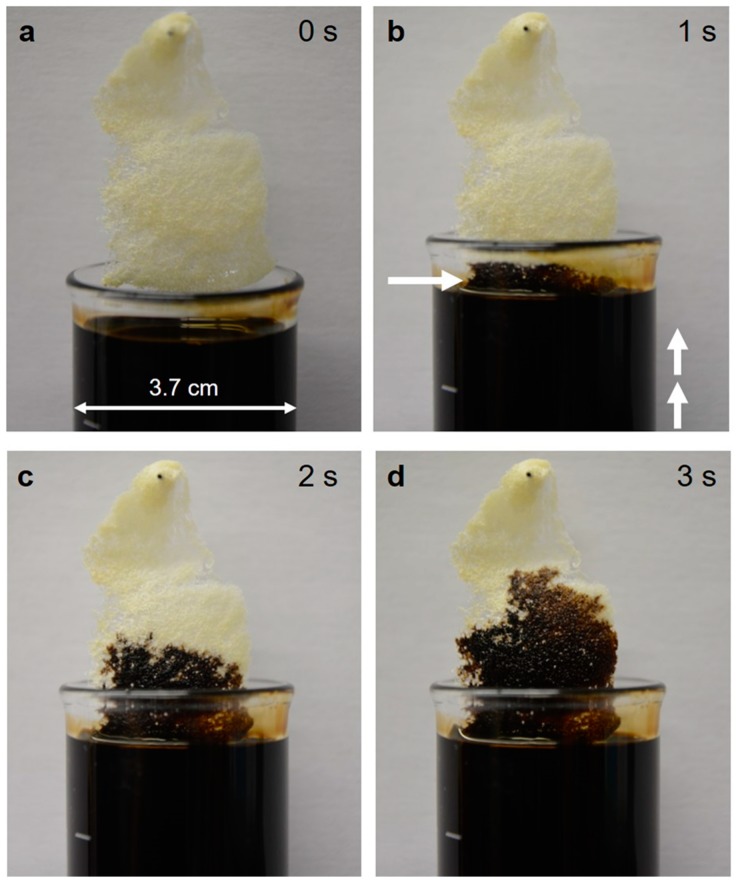
Capillary effect of the 3D chitinous microtubular scaffold isolated from *A. archeri* with respect to crude oil (**a**). On contact of the chitin with the surface of the oil (arrow, **b**), the latter is promptly distributed through the microtubular network (**b**,**c**,**d**). See also Figure 12.

In spite, of the hydrophilic nature of the purified (lipid- and wax-free) 3D chitin scaffold isolated from *A. archeri*, the chitin reacts immediately on coming into contact with the surface of the tested crude oil (Figure 11). The same effect was observed by the team of Bo Duan when comparing an artificial chitin sponge synthesized by a freeze-drying method with a modified hydrophobic surface [52]. It has also been reported that chitin has a low oil absorbance capacity, if there is competition between water and oil absorption [53,54]. The rapid uptake of oil may result from the capillary action of the 3D tubular network and the presence of van der Waals forces.

**Figure 12:**
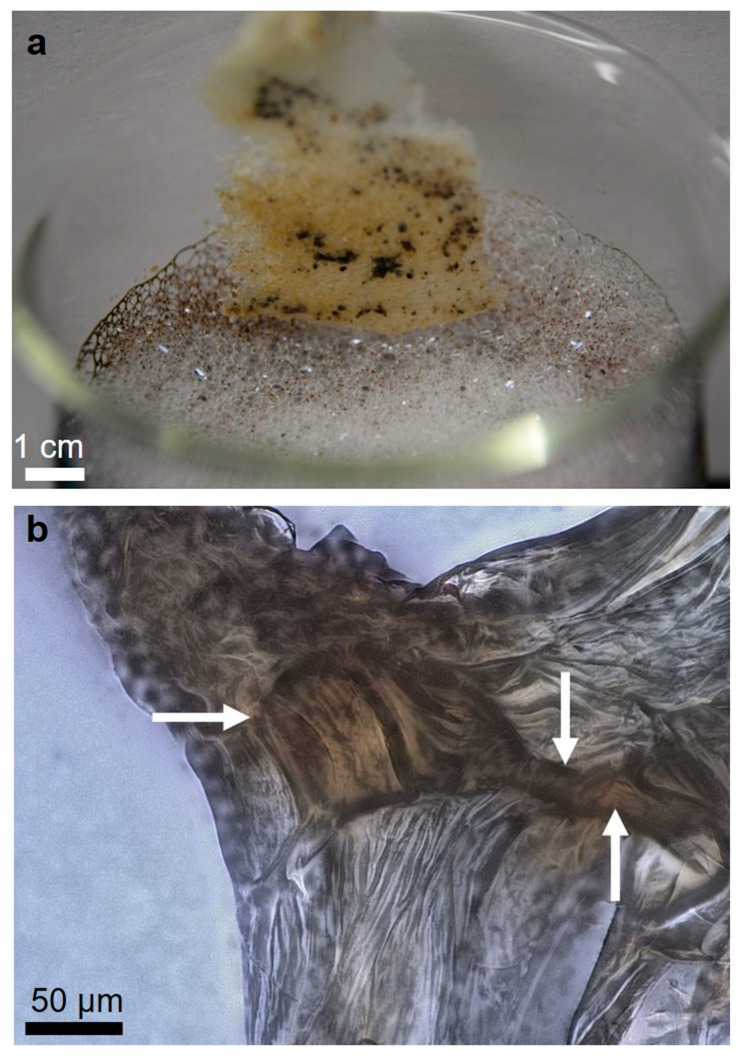
Rinsing of the chitin scaffold initially contaminated with crude oil (see Figure 11d) in soap solution promotes its removal from the surface of the scaffold (**a**). Nevertheless, residual oil is observable within the chitinous microtubes (arrows, **b**).

Rinsing the scaffold contaminated with crude oil (Figure 11d) in a soap solution at 40 °C removes the oil from the scaffold’s surface (Figure 12a). However, light microscopy observation of the inner surface of the microtubes of the chitinous scaffolds shows a significant presence of oil within those tubular structures (Figure 12b).

**Figure 13:**
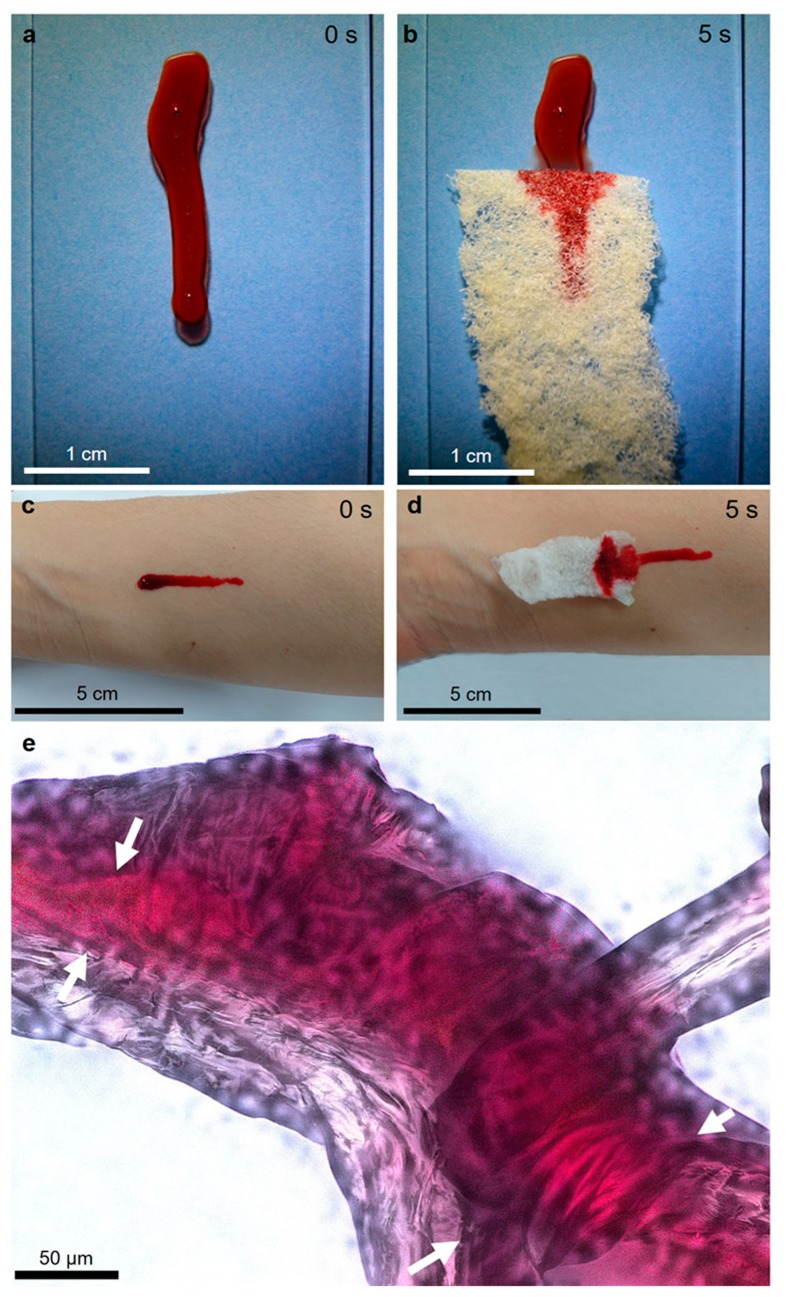
Pig blood as a model liquid is adsorbed by chitinous scaffold isolated from the *A. archeri* demosponge both from glass (**a**,**b**) and from a human skin surface (**c**,**d**). The blood remains confined within the axial channels (arrows) of the microtubular sponge chitin, even after rinsing with distilled water at room temperature (**e**).

We observed a similar effect when pig blood was used (Figure 13). Due to its red color, this blood, located within the axial channels of the anastomosing microtubes of the *A. archeri* chitin microfibers, is very visible (Figure 13e).

One possible application of 3D structured sponge-like hemostatic biomaterials could be as a compress during uncontrolled hemorrhaging (open wounds). It has been recognized that chitin of crustacean origin induces blood coagulation by adhering to platelets, forming a chitin/platelet complex that accelerates the polymerization of the fibrin monomer to form a blood clot (for review, see [55]). Consequently, several studies on chitin in the form of nanocrystals [56] or powders [55] as a potential hemostatic biomaterial have been carried out. However, for example, a hydrosol containing nanocrystalline particles showed no effect on the aggregation of human platelets and the clotting time of platelet-poor plasma in corresponding coagulation tests [56]. Therefore, we propose that experiments using 3D structured sponge chitins, including those from *A. archeri*, should be carried out to evaluate their hemostatic properties in future clinical settings.

**Figure 14:**
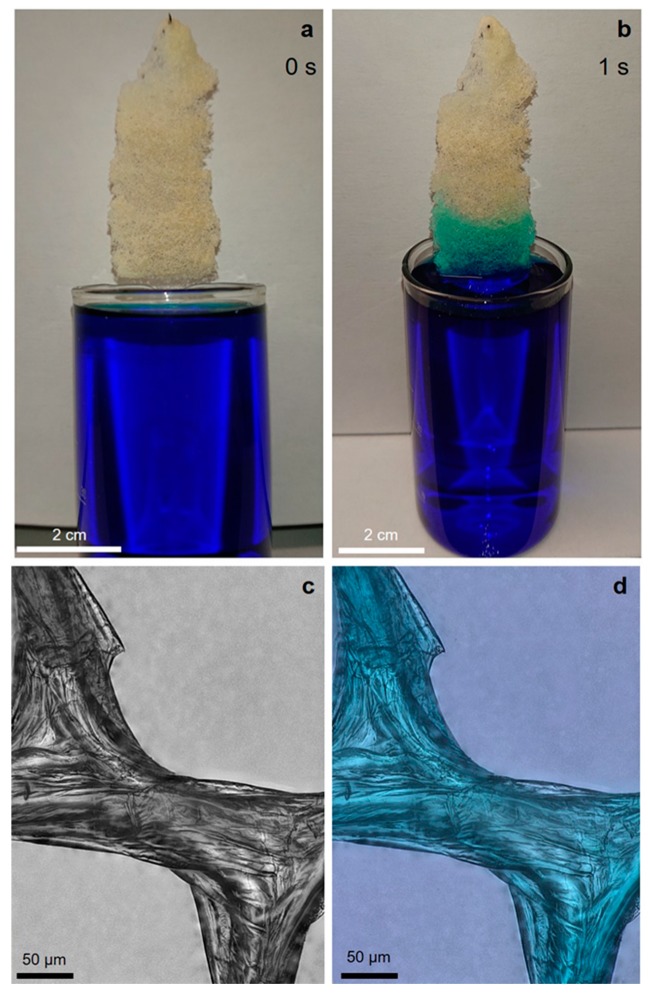
Image illustrating the adsorption of methylene blue (MB) on isolated 3D chitin scaffolds (**a**,**b**). The MB solution clearly reveals the tubular nature of the chitinous fiber in the scaffolds, resulting in the rising of liquid due to capillary forces. Light microscopy images of chitin after MB adsorption (**c**,**d**) clearly indicate that this dye is chemically adsorbed on the chitin surface as well as inside the chitinous microtubes, and cannot be removed from chitin by washing in hot distilled water.

Chitin has been considered recently as a biopolymer, which can be used as a low-cost and eco-friendly material for dye adsorption. Dotto et al. [57] reported ultrasonically-modified chitin as an effective sorbent for the removal of methylene blue (MB) from aqueous solutions by adsorption. Correspondingly, we have proven that 3D chitinous tubular scaffolds from *A. archeri*, obtained by the microwave-assisted method, can be used as a potential sorbent for MB. The swelling capacity of these scaffolds, with respect to water, was measured at 255 ± 8%. It should be noted that adsorption of the dye occurs on the surface as well as within the chitinous microtubes, and the dye could not be removed from the chitin by simply washing the chitin in hot distilled water (Figure 14). This confirms that the dye is chemically bonded to the chitin through electrostatic interactions with the chitin’s OH and *N*-acetyl groups [57]. The unique 3D morphology and open cell structure of the isolated chitin scaffold and its observed affinity to MB open the way to use such scaffolds as a new type of renewable filling for fixed bed reactors used for the purification of dye-contaminated wastewater.

## 3. Discussion

Microwave radiation has recently been shown to be a useful tool in modern green chemistry, with potential applications in a wide range of chemical processes [58,59]. Microwave-assisted extraction (MAE) is a green technique that offers numerous advantages, including the reduction of both extraction time and solvent consumption [60]. This method allows the acceleration of energy transfer, facilitating the solvation reaction and promoting the disruption of weak hydrogen bonds. It is currently considered a very attractive alternative to conventional extraction approaches (e.g., the Soxhlet method). Recently, microwave irradiation has been shown to be a promising technique for the extraction and modification of polysaccharides from natural sources [61], including chitin chemistry. This method is mostly used as an alternative for the preparation of chitosan from various chitinous sources [62,63,64,65]. It has been confirmed that microwave-assisted chitin processing reduces the time of deacetylation from ∼8 h [64] to 5.5 min [65]. The application of microwave irradiation reduces not only process duration, but also the quantities of energy and chemicals used for highly deacetylated chitosan production from crustacean shell waste from the food industry. Microwave-assisted chitin activation was patented by Peniston and Johnson in 1979 [66]. Those authors claimed that exposure to microwave energy for a period from about 1 to 30 min, for example, can result in greatly increased reactivity of the chitin. They selected a preferred microwave frequency of about 2450 MHz. While this is a frequency at which water is absorptive, it is far removed from frequencies causing resonance for water molecules. It thus appears that effects other than agitation of water molecules are responsible for the pronounced activation of the chitin. On this basis, we used a similar microwave frequency in our experiments with *A. archeri*. Interestingly, Wongpanit et al. [67] reported microwave treatment as an effective method for activation of carboxymethyl chitin and carboxymethyl chitosan. They showed that human fibroblasts adhered on the surface of microwave-treated CM-chitosan films much better than on the surface of microwave-treated CM-chitin films. Our contribution to this area of research is that both (i) morphologically defined chitinous scaffolds with highly organized architecture and (ii) pigments can be effectively isolated using microwave-assisted extraction after only 55 min of treatment, including rinsing procedures.

The obtained pigment-based extracts (see Figure 3) will be studied in our future work with regard to the identification of bromotyrosines typical for *A. archeri.* For example, Fistularin 3 and 11-ketofistularin 3, isolated previously from this sponge species, showed antiviral activity against feline leukemia [68]. Another bromotyrosine, known as *archerine*, exhibited antihistamine activity on the isolated guinea pig ileum at concentrations as low as 1 μM [69]. Because of the dark color of the isolated pigments it is possible that the extract may also contain uranidine [70]. Interestingly, the influence of alkaline treatment on the yield and molecular structure of bromotyrosines remains unknown.

Practical applications of demosponge chitinous scaffolds for uranium adsorption [71] for the development of supercapacitors [72] and hybrid composites [73,74], including extreme biomimetics approaches [75,76], as well as in tissue engineering [10,77,78,79], have previously been reported. Here, we describe, for the first time, the ability of 3D chitinous constructs isolated from *A. archeri* using an express method to swell with such substances as crude oil, pig blood, and MB dye in a few seconds, due to a capillary effect. This property may open the way to key applications of the unique and renewable sponge chitin in remediation, biomedicine, and wastewater treatment. One of the advantages of such chitin is that it can be quickly isolated as a ready-to-use microtubular scaffold.

Furthermore, a corresponding paper about mechanical properties of selected chitinous scaffolds isolated from diverse verongiid sponges using different methodological approaches is currently in preparation. In that work, we will also present data concerning crystallinity index, degree of acetylation, specific surface area (BET), Young modulus, etc. Obviously, we will present these data in comparison with chitin of crustacean and fungal origins.

One of the most promising applications of ready-to-use chitinous microtubular scaffolds of sponge origin is biomedicine. However, it should be noted that both natural as well synthetic biopolymer-based three-dimensional, open pore scaffolds have evolved as gold standards for biomedicine in recent applications, owing to their superior role in tissue regeneration [80]. Three-dimensional polymer networks have attracted significant attention owing to their “soft-and-wet” nature, which is similar to biological tissues. The major drawback of such scaffolds is their relatively poor mechanical strength [81]. However, several possible solutions can be used to overcome this drawback. Among them, we highlight the controlled remineralization of biopolymers with selected inorganic compounds (i.e., hydroxyapatite) [82] or silica [83,84]. The infiltration and crystallization of the mentioned inorganic phases on all three levels of the chitin’s hierarchical organization will definitively lead to the formation of a mechanically robust scaffold with superior performance in such advanced applications. Alternatively, to increase mechanical properties, biopolymeric scaffolds are often crosslinked. Inspired by the sclerotization of an insect cuticle, Chen et al. [85] reported a quinone crosslinking reaction for chitin hydrogels to increase their mechanical properties. The obtained crosslinked product shown a tenfold higher mechanical strength. Nevertheless, this is a topic for further studies. We believe that the present article will trigger scientific interest on the application of naturally pre-structured 3D scaffolds of sponge origin in biomedicine, and we believe that mechanical reinforcement of such scaffolds will soon be reported.

However, if the large-scale testing and application of chitin scaffolds is to be promoted, it will be necessary to secure the supply of such sponges via aquaculture [86], as wild harvest of these organisms will be not sustainable. Rohde and Schupp [87] have already demonstrated in a study of another Verongid sponge, *Ianthella basta*, that growth and regeneration rates in this sponge were sufficient to secure a supply for potential biomedical application. Further studies with other Verongiid sponges will be needed to establish this order of sponges as a viable source of chitin scaffolds and to enable the use of these naturally prefabricated constructs in biomedical applications.

## 4. Materials and Methods

### 4.1. Sample Collection

Samples of the demosponge, *Aplysina archeri* (Higgin 1875), were collected at depths of 10–25 m by scuba divers around the Caribbean islands of Saint Vincent and Curaçao in May 2015 and June 2017, during the Pacotilles expedition. All permits that were required for the described study were obtained during this expedition and complied with all relevant regulations. The species was identified by A. Ereskovsky.

### 4.2. Isolation of Chitin

#### 4.2.1. Modified Standard Method

The isolation of the chitin scaffold from *A. archeri* was performed by a modification of the well-known standard method [4]. Firstly, the organic material was extracted with 2.5 M NaOH (Th. Geyer GmbH & Co. KG, Renningen, Germany,) at 37 °C for 6 h. Next, the resulting mineral skeletons of the sponges were treated with 2.5 M NaOH at 37 °C for 18 h. They were then washed with distilled water. The neutralized skeleton was then treated with 20% acetic acid (Th. Geyer GmbH & Co. KG, Renningen, Germany) at 37 °C for 6 h and neutralized by washing with distilled water. Alkaline and acidic extraction was repeated until the skeleton was completely demineralized, transparent, and soft (114 h).

#### 4.2.2. Microwave-Assisted Approach for Chitin Isolation

The sample material of *A. archeri* was cut into pieces 5 cm × 2 cm in size and used for chitin isolation. The isolation procedure is presented schematically in Figure 3. Briefly, in step I the residual water-soluble salts were removed by pretreatment of sponge skeletons in distilled water using microwave irradiation (MWI) for 3 min. In step II, the sample was treated with 1% NaOH and irradiated with microwaves for 2 min. The 3D scaffolds were then carefully isolated and rinsed three times with distilled water to remove and separate residual red-brownish pigments. In step III, the 3D scaffolds were treated with 20% acetic acid under MWI for 1 min and were then washed in distilled water (up to pH 6.8). In step IV, samples were decolorized using H_2_O_2_ treatment at pH~10 under MWI for 1 min and then washed with distilled water until a neutral pH was reached.

Hydrogen peroxide was chosen for bleaching because it is reported that hydrogen peroxide in a microwave field, under the conditions of the experiment, does not cause significant changes in the chemical structure of the polymer [88]). Steps II–IV were repeated four times to obtain pure, colorless, 3D chitinous scaffolds.

#### 4.2.3. Fluorescent Microscopy Analysis

The isolated chitin fibers were observed using a BZ-9000 instrument (Keyence, Osaka, Japan) in fluorescent and light microscopy mode.

#### 4.2.4. Calcofluor White Staining

Upon binding with polysaccharides, calcofluor white (CFW) enhanced their fluorescence. The isolated fibers from the selected *A. archeri* skeletons were investigated with light and fluorescent microscopy before and after staining with CFW (Fluorescent Brightener M2R, Sigma-Aldrich). For staining, 20 µL of a solution of 10 g glycerin and 10 g NaOH in 90 mL water was applied. After 15 s, the CFW was added and kept for 30 min in darkness at room temperature. The fibers were washed with distilled water to remove unattached CFW and then dried at room temperature.

#### 4.2.5. Chitinase Digestion Test

To observe the chitinase digestion process of the fibers isolated from sponge skeletons, the BZ-9000 instrument was used in light and fluorescent microscopy mode. Single fibers were rinsed with 50 mL phosphate buffer (pH 6.0). After the removal of the phosphate buffer, chitinase from *Streptomyces griseus* (EC 3.2.1.14, No. C-6137, Sigma-Aldrich) was added. In the following 10 h, the microscope documented the digestion of the fibers by taking light field and fluorescent images every 30 min.

#### 4.2.6. ATR-FTIR Spectroscopy

Infrared spectroscopy was used for qualitative analysis of the isolated fibers. ATR-FTIR (attenuated total reflectance Fourier transformation infrared spectroscopy) spectra were recorded with the Nicolet 380 FTIR spectrometer (Thermo Scientific, Inc., Madison, WA, USA). The spectra were analyzed with appropriate software (OMNIC Lite Software, Madison, WA, USA). In addition to the spectra of the fiber material, spectra of the *a*-chitin standard (INTIB GmbH, Freiberg, Germany) and a chitosan standard (Sigma-Aldrich) were recorded. Postprocessing of the recorded spectra was performed with OriginLab 2015.

#### 4.2.7. Raman Spectroscopy

Raman spectra were recorded with a Raman spectrometer (Raman Rxn1^TM^, Kaiser Optical Systems Inc., Ann Arbor, MI, USA) coupled with a light microscope (DM2500 P, Leica Microsystems GmbH, Wetzlar, Germany). The laser beam had a power of 400 mW, which resulted in 110 mW at the sample due to the transmission of the microscope optics. The spectral range was from 200 to 3250 cm^−1^, with a set resolution of 4 cm^−1^. For improvement of the signal-to-noise ratio, the integration time was set to 1 s and an addition of 50 spectra was performed. The baseline was implemented with MATLAB (MathWorks Inc., Natick, MA, USA) and further postprocessing was carried out with OriginPro 2015.

#### 4.2.8. Scanning Electron Microscopy

Scanning electron microscopy was performed using an XL 30 ESEM Philips-Scanning Electron Microscope (FEI Company, Peabody, MA, USA). Samples were fixed on a sample holder with carbon patches, and these were then covered with carbon or with a 5–10 µm gold layer using an Edwards Sputter Coater S150B (BOC Edwards, Wilmington, MA, USA).

#### 4.2.9. EDX

The elements were analyzed by energy dispersive X-ray spectroscopy in the EDX analysis system from EDAX (Mahwah, NJ, USA) and XL 30 ESEM Philips-Scanning Electron Microscope (FEI Company, Peabody, MA, USA).

#### 4.2.10. Electrospray Ionisation Mass Spectrometry (ESI-MS) 

Samples were prepared for ESI-MS as follows. The isolated chitin scaffold was hydrolyzed in 6M HCl for 24 h at 90 °C. The resulting solution was filtered with a 0.4-micron filter, and the filtrate was freeze-dried to remove excess HCl. The solid residue was dissolved in water for ESI-MS analysis. Standard d-glucosamine, used as a control, was purchased from Sigma (Sigma-Aldrich, Taufkirchen, Germany). All ESI-MS measurements were performed on an Agilent Technologies 6230 TOF LC/MS spectrometer (Applied Biosystems, Foster City, CA, USA). Nitrogen was used as a nebulizing and desolvation gas. Graphs were generated using Origin 8.5 for PC.

#### 4.2.11. Sorption Experiments

For the sorption experiments, crude oil (collected using sterile glass bottles from the oilfield located in Borislav, Ukraine), pig blood (Südost-Fleisch GmbH, Altenburg, Germany) and methylene blue (Sigma-Aldrich) were used.

#### 4.2.12. Swelling Capacity Measurements

The swelling capacity (water) of isolated 3D chitinous scaffolds was calculated using the following formula [89]:

$$Swelling\;capacity\;\left(\%\right)=\frac{m_{S}-m_{d}}{m_{d}}\cdot 100\%$$

where, *m_S_* is the mass of the swollen sponge chitin and *m_d_* is the mass of the dry sponge chitin.

## 5. Conclusions

In this study, the presence of chitin in the skeleton of *A. archeri* has been proven for the first time. Furthermore, microwave-assisted demineralization and deproteinization have been established as new and rapid methods for both chitin and pigment extraction from verongiid demosponges. It has been demonstrated, beyond a doubt, that the new approaches enable a significant improvement in the time efficiency of chitin isolation from Verongiida sponges without destruction of the unique fibrous interconnected structure of the isolated biological material. Furthermore, these mechanically stressed fibers still have the capacity for saturation with water, methylene blue dye, crude oil, and blood, which will be crucial for future applications of such naturally prefabricated 3D chitinous centimeter-sized scaffolds in diverse technological and biomedical fields. As the analysis of the isolated scaffolds has shown, under the conditions studied here, the microwaves do not deacetylate the chitin to chitosan. The method proposed in this study both reduced the consumption of the aggressive chemicals as well as drastically reduced the time of chitin extraction. Therefore, microwave assisted extraction is a viable, cost efficient, and fast method for the production of chitin scaffolds from marine sources.
